# miR-149-3p Is a Potential Prognosis Biomarker and Correlated with Immune Infiltrates in Uterine Corpus Endometrial Carcinoma

**DOI:** 10.1155/2022/5006123

**Published:** 2022-06-08

**Authors:** Xiaoyuan Lu, Li Jing, Sicong Liu, Haihong Wang, Buze Chen

**Affiliations:** ^1^Department of Gynecology, The Affiliated Hospital of Xuzhou Medical University, Xuzhou 221000, Jiangsu, China; ^2^Xuzhou Medical University, Xuzhou 221000, Jiangsu, China; ^3^Graduate School, Xuzhou Medical University, Xuzhou 221000, Jiangsu, China

## Abstract

**Background:**

Endocrine disruption is an important factor in the development of endometrial cancer. Expression of miR-149-3p is observed in some cancer types, while its role in uterine corpus endometrial carcinoma (UCEC) is unclear.

**Methods:**

The clinical and genomic data and prognostic information on UCEC were obtained for patients from the TCGA database. The Kruskal–Wallis test, Wilcoxon signed-rank test, and logistic regression were used to analyze the relationship between clinical characteristics and miR-149-3p expression. Kaplan–Meier survival curve analysis was used to study the influence of miR-149-3p expression and miR-149-3p target genes on the prognosis of UCEC patients. The TargetScan, PicTar, Gene Ontology (GO), and Kyoto Encyclopedia of Genes and Genomes (KEGG) analyses were used to determine the involvement of miR-149-3p target genes in function. Immune infiltration analysis was used to analyze the functional involvement of miR-149-3p. QRT-PCR was used to validate the expression of miR-149-3p in UCEC cell lines.

**Results:**

High expression of miR-149-3p in UCEC was significantly associated with age (*P* < 0.001), histological type (*P* < 0.001), histological grade (*P* < 0.001), tumor invasion (*P*=0.014), and radiation therapy (*P*=0.011). High miR-149-3p expression predicted poorer overall survival (OS) (HR: 2.56; 95% CI: 1.64–4.00; *P* < 0.001), progression-free interval (PFI) (HR: 1.85; 95% CI: 1.29–2.65; *P*=0.001), and disease-specific survival (DSS) (HR: 2.33; 95% CI: 1.37–3.99; *P*=0.002). Low expressions of miR-149-3p target genes, including ADCYAP1R1, CGNL1, CHST3, CYGB, DNAH9, ESR1, HHIP, HIC1, HOXD11, IGF1, INMT, LSP1, MTMR10, NFIC, PLCE1, RARA, SNTN, SPRYD3, and ZBTB7A, were associated with poor OS in UCEC. MiR-149-3p may be involved in the occurrence and development of UCEC via pathways including PI3K-Akt signaling pathway, Ras signaling pathway, AGE-RAGE signaling pathway in diabetic complications, focal adhesion, and MAPK signaling pathway. miR-149-3p may inhibit the function of CD8 T cells, cytotoxic cells, eosinophils, iDC, mast cells, neutrophils, NK CD56bright cells, NK CD56dim cells, pDC, T cells, T helper cells, TFH, Th17 cells, and Treg. miR-149-3p was significantly upregulated in UCEC cell lines compared with endometriotic stromal cells.

**Conclusion:**

High expression of miR-149-3p was significantly associated with poor survival in UCEC patients. It may be a promising biomarker of prognosis and response to immunotherapy for UCEC patients.

## 1. Introduction

Uterine corpus endometrial carcinoma (UCEC) is one of the three main gynaecological malignancies, the incidence of which increases over time. Endometrial cancer is an epithelial tumor of the endometrium and a malignant tumor of the female reproductive system with a high incidence, posing a serious threat to the health of women worldwide [1, 2]. It currently ranks second in the incidence of gynaecological cancers in developing countries, behind cervical cancer [3]. According to the pathogenesis and biological behavior characteristics of endometrial cancer, endometrial cancer can be divided into estrogen-dependent (type I) and nonestrogen-dependent (type II), and most endometrial cancers belong to the former, which means endometrial cancer is closely related to endocrine. The etiological mechanisms of UCEC have not been fully elucidated. Risk factors for UCEC that have been identified include unstable postmenopausal or perimenopausal oestrogen levels, obesity, infertility, diabetes, hypertension, and family history [4]. Early clinical signs in patients with UCEC include irregular vaginal bleeding in the postmenopausal or perimenopausal period, pelvic cramps, and abdominal pain. The current lack of effective early diagnosis of UCEC has resulted in many UCEC patients missing the best time for treatment, and the poor prognosis of UCEC patients, especially those with metastases after surgery and radiotherapy, is also one of the important issues to be addressed in the current treatment of UCEC [5]. Although progress has been made in early detection and treatment, a considerable number of advanced UCEC cases have been diagnosed [6, 7]. With the development of gene microarray technology, increasing research data are available to help resolve the complex pathogenesis of UCEC and monitor the disease progress. Therefore, systematic bioinformatic analysis of UCEC-related disease data allows for a rapid search for biomarkers that can be used for UCEC prognosis.

MicroRNAs (miRNAs) are a unique class of endogenous and small noncoding RNAs that are approximately 18 to 25 nucleotides in length. They alter gene expression at the posttranscriptional level primarily through complete or incomplete base pairing with the 3′ untranslated region (3′UTR) of their target mRNAs. Translational repression and mRNA degradation are the 2 main pathways through which miRNAs direct gene regulation [8]. Many studies have shown that different miRNAs are abnormally expressed in different tumors and participate in tumor formation and growth as oncogenes or oncogenes [9]. There is substantial evidence that miRNAs are stably detectable in serum and plasma and have the potential to be noninvasive biomarkers for the diagnosis and prognosis of various cancers. miRNAs offer an innovative idea for screening and detection of cancer patients [10–12].

In oral squamous cell carcinoma, reduced levels of miRNA-149-3p lead to malignant progression and predict poor prognosis [13]. Increased miR-149-3p expression significantly inhibited the proliferation, migration, and invasion of bladder cancer cells [14]. Only a few studies mentioned that the lnc HOXB-AS1 was upregulated in endometrial cancer and spongy miR-149-3p upregulated Wnt10b [15]. However, the expression of miR-149-3p in UCEC and its relation to clinical features has not been well studied.

The present study examined the expression of miR-149-3p in UCECs using an online database and analyzed the relationship between expression levels and clinical characteristics. A survival curve was drawn to analyze the relationship between miR-149-3p expression level and overall survival (OS). Important contributions of miR-149-3p target genes to function were identified by TargetScan, PicTari, Gene Ontology (GO), and Kyoto Gene and Genome Encyclopedia (KEGG) analyses. The functionally significant involvement of miR-149-3p was analyzed by immune infiltration analysis. QRT-PCR was used to validate the expression of miR-149-3p in UCEC cell lines. The results of this study could provide new prognostic biomarkers for UCEC.

## 2. Patients and Methods

### 2.1. Clinical Information

The analysis was carried out according to references [16, 17]. R (version 3.6.3) was used for statistical analysis and visualization. The R package was the base R package. The molecule was hsa-miR-149-3p (MIMAT0004609). The grouping condition was median. The disease was UCEC. Data were obtained from miRNAseq data from level 3 BCGSC miRNA Profiling in the TCGA (https://portal.gdc.cancer.gov/) UCEC project. The miRNAseq data in RPM (reads per million mapped reads) format were log2-transformed.

### 2.2. Differential Expression of miR-149-3p

 
*Unpaired Samples*. The analysis was carried out according to reference [18]. R (version 3.6.3) was used for statistical analysis and visualization. ggplot2 (version 3.3.3) was used for visualization. The molecule was hsa-miR-149-3p. The disease was UCEC. Data were miRNAseq data from level 3 BCGSC miRNA Profiling in TCGA UCEC. The miRNAseq data in RPM format were log2 transformed. Data were unfiltered.
 
*ROC Curves*. The analysis was carried out according to reference [18]. R (version 3.6.3) was used for statistical analysis and visualization. The R packages were the pROC package (version 1.17.0.1) (for analysis) and the ggplot2 package (version 3.3.3) (for visualization). The molecule was hsa-miR-149-3p. The clinical variable is normal versus tumor. The disease was endometrial cancer. Data were miRNAseq data from level 3 BCGSC miRNA Profiling in the TCGA UCEC project. The miRNAseq data in RPM format were log2 transformed. Data are unfiltered.

### 2.3. Correlation of miR-149-3p Expression with Clinical Features

Correlation analysis of gene expression with clinical characteristics was carried out according to reference [16]. R (version 3.6.3) was used for statistical analysis and visualization. ggplot2 (version 3.3.3) was used for visualization. The molecule was hsa-miR-149-3p. Clinical variables were age, histological type, tumor invasion, and radiation therapy. The disease was UCEC. Data were miRNAseq data and clinical data from level 3 BCGSC miRNA Profiling in the TCGA UCEC project. The miRNAseq data in RPM format were log2 transformed. Data filtering conditions included removing controls/normal (not all items have controls/normal) and keeping clinical information available.

Logistics analysis was carried out according to references [18, 19]. R (version 3.6.3) was used for statistical analysis and visualization. R package was mainly a basic package. The statistical method was a dichotomous logistic model. The independent variable was hsa-miR-149-3p. The type of independent variable was low high dichotomous. The disease was UCEC. Data were miRNAseq data from level 3 BCGSC miRNA Profiling in the TCGA UCEC project. The miRNAseq data in RPM format were log2 transformed. Data filtering conditions include removal of control/normal (not all items have control/normal) and retention of clinical information.

### 2.4. Prognostic Value of miR-149-3p Expression in UCEC Patients

The Kaplan–Meier method analysis was carried out according to references [16, 17]. R (version 3.6.3) was used for statistical analysis and visualization. The survminer package (version 0.4.9) was used for visualization. The survival package (version 3.2-10) was used for statistical analysis of survival data. The molecule was hsa-miR-149-3p. Subgroups were 0–50 and 50–100. Prognostic types were OS, PFI, and DSS. The disease was UCEC. Data were miRNAseq data from level 3 BCGSC miRNA Profiling in the TCGA UCEC project. The miRNAseq data in RPM format were log2 transformed. Supplementary data were prognostic data from reference [20]. Data filtering conditions included removal of control/normal (not all items had control/normal) and retention of clinical information.

COX regression analysis was carried out according to references [19–21]. R (version 3.6.3) was used for statistical analysis and visualization. The survival package (version 3.2-10) was used for statistical analysis of survival data. Statistical method was the Cox regression module. The prognosis type was OS. Included variables were clinical stage, primary therapy outcome, age, weight, height, BMI, histological type, residual tumor, histologic grade, tumor invasion (%), menopause status, hormones therapy, diabetes, radiation therapy, surgical approach, and hsa-miR-149-3p. The disease was UCEC. Data were miRNAseq data from level 3 BCGSC miRNA Profiling in the TCGA UCEC project. The miRNAseq data in RPM format were log2 transformed. Supplementary data were prognostic data from reference [20]. Data filtering included removal of control/normal (not all items have control/normal) and retention of clinical information.

Forest plot: software was R (version 3.6.3). R package was the ggplot2 package.

Nomogram plot analysis was carried out according to literature [19, 20]. R package was rms package and survival package. The prognosis type was OS. Included variables were clinical stage, primary therapy outcome, age, histological type, residual tumor, tumor invasion, radiation therapy, and miR-149-3p.

### 2.5. Predicted Putative Targets of miR-149-3p

miR-149-3p targets were obtained from Database TargetScan, miRanda, TarBase, miRTarBase, miR2Disease, miRecords, and miRWalk [22–25]. UCEC mRNA expression media files are downloaded from the website (https://bioinfo.life.hust.edu.cn/miR_path/download.html). The UCEC prognosis-related genes were analyzed according to reference [16]. R (version 3.6.3) was used for statistical analysis and visualization. The survminer package (version 0.4.9) was used for visualization. The survival package (version 3.2-10) was used for statistical analysis of survival data. The statistical method was Cox. The subgroups were 0–50 versus 50–100. The prognosis type was OS. The disease was UCEC. Data were miRNAseq data from level 3 BCGSC miRNA Profiling in the TCGA UCEC project. The miRNAseq data were log2 transformed in RPM format. Supplementary data were prognostic data from reference [20]. Data filtering criteria included removal of control/normal (not all items had control/normal) and retention of clinical information. The common genes among miR-149-3p target genes, UCEC-downregulated genes, and UCEC prognosis-related genes were analyzed according to the Venn diagram.

### 2.6. Relationship between miR-149-3p Target Gene Expression and Prognosis

The analysis was performed according to reference [18]. R (version 3.6.3) was used for statistical analysis and visualization. The survminer package (version 0.4.9) was used for visualization. The survival package (version 3.2-10) was used for statistical analysis of survival data. Molecules were ADCYAP1R1, CGNL1, CHST3, CYGB, DNAH9, ESR1, HHIP, HIC1, HOXD11, IGF1, INMT, LSP1, MTMR10, NFIC, PLCE1, RARA, SNTN, SPRYD3, and ZBTB7A. Subgroups were 0–50 and 50–100. Prognostic types were OS, PFI, and DSS. The disease was UCEC. Data were RNAseq data in level 3 HTSeq-FPKM format from the TCGA UCEC project. RNAseq data in FPKM format were converted to TPM (transcripts per million reads) format and log2 transformed. Supplementary data were prognostic data from reference [20]. Data filtering conditions included removal of control/normal (not all items had control/normal) and retention of clinical information.

### 2.7. GO and KEGG Analyses of miR-149-3p Targets

The Database for Annotation, Visualization, and Integrated Discovery (DAVID) can provide a comprehensive set of functional annotation tools to facilitate understanding of the biological significance behind a large number of genes. GO and KEGG analyses were performed on the targets of miR-149-3p using the DAVID database (https://david.ncifcrf.gov/) [26–28]. GO and KEGG enrichment pathways (adjusted *P* value less than 0.05) were considered significant categories.

### 2.8. Immune Infiltration Analysis by ssGSEA

The analysis was performed according to reference [18]. R (version 3.6.3) was used for statistical analysis and visualization. The R package was the GSVA package (version 1.34.0) [29]. The immunoinfiltration algorithm was ssGSEA (built-in algorithm of the GSVA package). The molecule was hsa-miR-149-3p. Immune cells were aDC (activated DC), B cells, CD8 T cells, cytotoxic cells, DC, eosinophils, iDC (immature DC), macrophages, mast cells, neutrophils, NK CD56bright cells, NK CD56dim cells, NK cells, pDC (plasmacytoid DC), T cells, T helper cells, Tcm (T central memory), Tem (T effector memory), Tfh (T follicular helper), Tgd (T gamma delta), Th1 cells, Th17 cells, Th2 cells, and Treg. The disease was endometrial cancer. Data were miRNAseq data from level 3 BCGSC miRNA Profiling in the TCGA UCEC project. The miRNAseq data in RPM format were log2 transformed. The data filtering condition was to remove control/normal (not all items had control/normal). Markers for 24 immune cells were obtained from reference [30].

### 2.9. QRT-PCR

Human endometriotic stromal cells (ESC) have been isolated from endometriotic tissue. Human UCEC cells Ishikawa and KLE were obtained from our laboratory. KLE cells were grown in F12. Ishikawa cells were grown in RPMI-1640. 10% fetal bovine serum was added to the medium to maintain the cell state. Add 1% antibiotics to the soil to prevent contamination. Cells were grown in a 37°C incubator containing 5% CO_2_. The miR-149-3p levels in the ESC, Ishikawa, and KLE cell lines were identified by qRT-PCR. The specific steps were performed according to reference [31]. The primer sequences used are as follows: U6, Forward: CTCGCTTCGGCAGCACA, Reverse: AACGCTTCACGAATTTGCGT; miR-149-3p, Forward: 5′-ACAGGGGAGGGACGGGGG-3′, Reverse: 5′-CAGTGCAGGGTCCGAGGTATT-3′.

### 2.10. Statistical Analysis

Statistical analysis was carried out according to reference [18]. All statistical analyses were performed using R (v.3.6.3). The Wilcoxon rank-sum test, chi-square test, and Fisher exact test were used to analyze the relationship between clinical characteristics and miR-149-3p. *P* values less than 0.05 were considered statistically significant.

## 3. Results

### 3.1. Clinical Characteristics

A total of 546 patients were analyzed in the present study (Table 1). The clinical stage included 341 Stage I (62.5%), 49 Stage II (9%), 128 Stage III (23.4%), and 28 Stage IV (5.1%). The primary therapy outcome included 20 PD (4.2%), 6 SD (1.3%), 12 PR (2.5%), and 437 CR (92%). The race included 20 Asian (4%), 109 Black or African American (21.8%), and 372 White (74.3%). The age included 205 patients (≤60, 55.5%) and 338 patients (>60, 44.5%). The height included 244 patients (≤160) (47%) and 275 patients (>160) (53%). The weight included 240 patients (≤80) (45.9%) and 283 patients (>80) (54.1%). The BMI included 209 patients (≤30) (40.6%) and 306 patients (>30) (59.4%). The histological type included 409 endometrioid (74.9%), 24 mixed (4.4%), and 113 serous (20.7%). The residual tumor included 371 *R*0 (90.9%), 22 *R*1 (5.4%), and 15 *R*2 (3.7%). The histologic grade included 98 *G*1 (18.3%), 121 *G*2 (22.6%), and 316 *G*3 (59.1%). Tumor invasion (%) included 260 patients (<50) (55.3%) and 210 patients (≥50) (44.7%). The menopause status included 34 pre (6.8%), 17 peri (3.4%), and 449 post (89.8%). The hormone therapy included 297 no (86.6%) and 46 yes (13.4%). The diabetes included 323 no (72.6%) and 122 yes (27.4%). The radiation therapy included 278 no (53.4%) and 243 yes (46.6%). The surgical approach included 207 minimally invasive (39.6%) and 316 open (60.4%). The age range was 57 to 71 years, with a median of 64 years.

### 3.2. miR-149-3p Expression is Correlated with Poor Clinical Characteristics of UCEC

**Figure 1:**
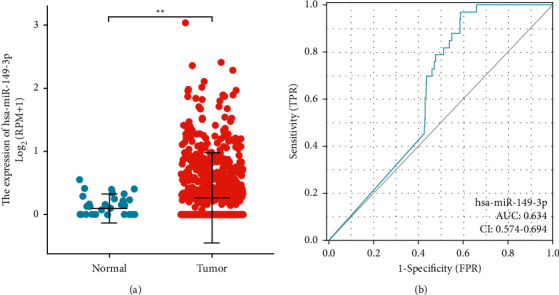
Expression of miR-149-3p in 537 UCEC and 33 normal tissues. (a) miR-149-3p relative expression levels (expressed in transcripts per million, TPM) were significantly higher in UCEC tissues (3.469 ± 0.0805) compared with normal tissues (2.70 ± 0.151). (b) ROC. Significance markers: ^*∗∗*^*P* < 0.01.

Genomic data were screened from the TCGA database to obtain data for 546 UCEC tissues and 33 normal tissue samples. miR-149-3p expression level in UCEC tissues (0.427 ± 0.515, *n* = 546) was significantly higher than that in normal tissues (0.135 ± 0.159, *n* = 33) (*P*=0.007) (Figure 1(a)). The area under curve (AUC) of miR-149-3p was 0.634 (Figure 1(b)), suggesting that miR-149-3p could be used as a promising biomarker to differentiate UCEC from nontumor tissue.

**Figure 2:**
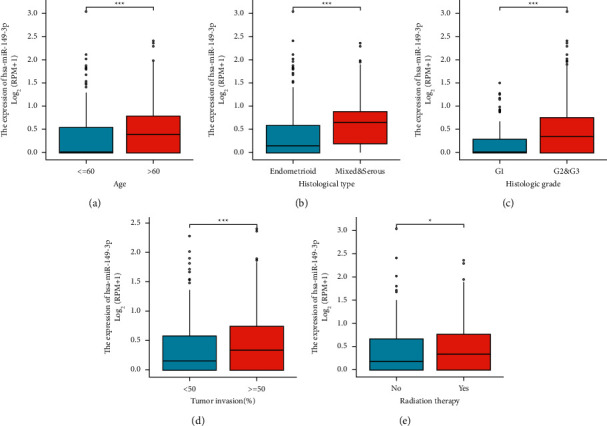
Association with miR-149-3p expression and clinical stage in UCEC. (a) Age, (b) histological grade, (c) histological grade, (d) tumor invasion, and (e) radiation therapy. Significance markers: ^*∗*^*P* < 0.05; ^*∗∗∗*^*P* < 0.001.

The characteristics of UCEC patients are shown in Table 1, in which 546 primary UCEC with both clinical and gene expression data were collected from the TCGA database. According to the mean value of the relative miR-149-3p expression, the patients with UCEC were divided into high (*n* = 273) and low (*n* = 273) expression groups. As shown in Figure 2 and Table 1, miR-149-3p was significantly related to age (*P* < 0.001), histological type (*P* < 0.001), histological grade (*P* < 0.001), tumor invasion (*P*=0.014), and radiation therapy (*P*=0.011). As shown in Table 2, miR-149-3p was significantly related to primary therapy outcome (HR: 0.501; 95% CI: 0.247–0.983; *P*=0.048), age (HR: 2.006; 95% CI: 1.411–2.863; *P* < 0.001), histological type (HR: 0.311; 95% CI: 0.204–0.470; *P* < 0.001), histologic grade (HR: 0.302; 95% CI: 0.183–0.485; *P* < 0.001), tumor invasion (HR: 1.608; 95% CI: 1.116–2.324; *P* < 0.011), and radiation therapy (HR: 1.595; 95% CI: 1.129–2.259; *P*=0.008).

### 3.3. Role of miR-149-3p in UCEC Patient Survival

**Figure 3:**
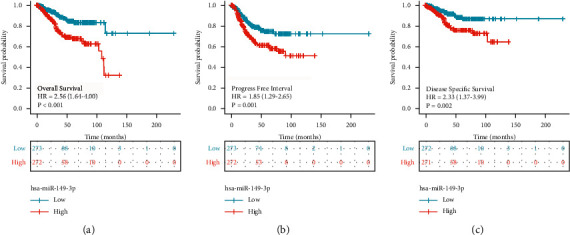
The relationship between the expression level of miR-149-3p and prognosis. (a) OS, overall survival; (b) PFI, progress-free interval; (c) DSS, disease survival specific.

**Figure 4:**
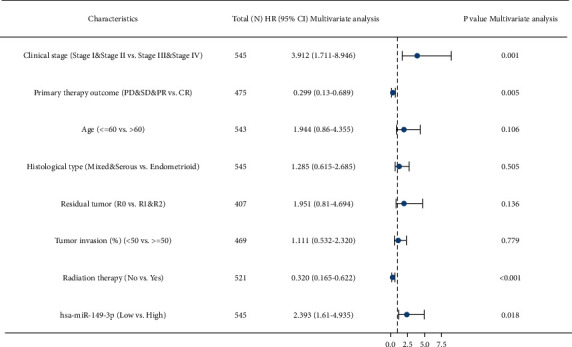
UCEC for multivariate Cox regression analysis of forest plots.

**Figure 5:**
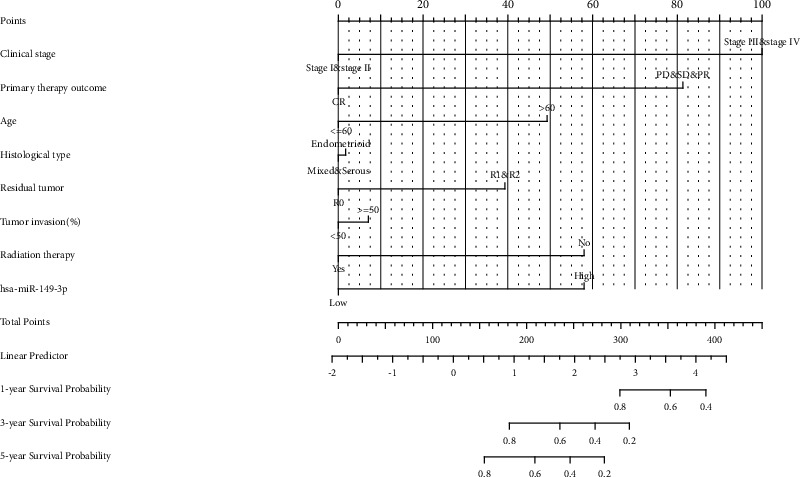
Nomogram predicts 1-, 3-, and 5-year overall survival in patients with UCEC.

The association between miR-149-3p expression and OS of patients with UCEC was evaluated by Kaplan–Meier analysis, which indicated that the expression of miR-149-3p was correlated with poor OS (HR: 2.56; 95% CI: 1.64–4.00; *P* < 0.001), PFI (HR: 1.85; 95% CI: 1.29–2.65; *P*=0.001), and DSS (HR: 2.33; 95% CI: 1.37–3.99; *P*=0.002) of UCEC patients (Figure 3). The above data indicated that miR-149-3p is a prognostic factor, and high miR-149-3p level is associated with poor OS. As shown in Table 3, high expression levels of miR-149-3p were associated with worse OS (HR: 2.56; 95% CI: 1.64–4.00; *P* < 0.001), clinical stage (HR: 3.774; 95% CI: 2.484–5.732; *P* < 0.001), primary therapy outcome (HR: 0.125; 95% CI: 0.075–0.208; *P* < 0.001), age (HR: 2.002; 95% CI: 1.236–3.242; *P*=0.005), histological type (HR: 0.366; 95% CI: 0.241–0.557; *P* < 0.001), residual tumor (HR: 3.081; 95% CI: 1.725–5.503; *P* < 0.001), histologic grade (HR: 22.795; 95% CI: 3.127–163.794; *P* < 0.001), tumor invasion (HR: 3.124; 95% CI: 1.909–5.112; *P* < 0.001), and radiation therapy (HR: 0.586; 95% CI: 0.375–0.914; *P*=0.018). As shown in Table 3 and Figure 4, clinical stage (HR: 3.774; 95% CI: 2.484–5.732; *P* < 0.001), primary therapy outcome (HR: 0.125; 95% CI: 0.075–0.208; *P* < 0.001), radiation therapy (HR: 3.774; 95% CI: 2.484–5.732; *P* < 0.001), and hsa-miR-149-3p (HR: 0.125; 95% CI: 0.075–0.208; *P* < 0.001) were independently correlated with OS in multivariate analysis. The above data indicated that miR-149-3p is a prognostic factor, and increased miR-149-3p is associated with poor OS. A nomogram was constructed to predict the 1-, 3-, and 5-year survival probability of UCEC patients by combing the expression of miR-149-3p with clinical variables, as shown in Figure 5.

### 3.4. Relationship between miR-149-3p Target Genes and Survival of UCEC Patients

**Figure 6:**
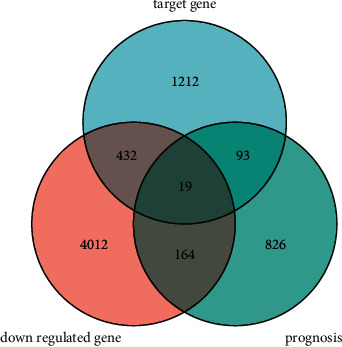
Venn diagram of common genes for miR-149-3p target genes, UCEC-downregulated genes, and prognosis-related genes.

There were 1756 miR-149-3p target genes, 4627 UCEC-downregulated genes, and 1102 UCEC prognosis-related genes in Figure 6. As shown in Figure 4 and Table 4, the 19 common genes included ADCYAP1R1, CGNL1, CHST3, CYGB, DNAH9, ESR1, HHIP, HIC1, HOXD11, IGF1, INMT, LSP1, MTMR10, NFIC, PLCE1, RARA, SNTN, SPRYD3, and ZBTB7A.

### 3.5. GO and KEGG Analyses of miR-149-3p Target Genes

**Figure 7:**
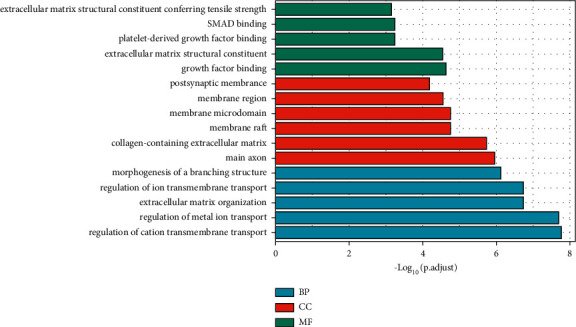
GO analysis of miR-149-3p target genes.

**Figure 8:**
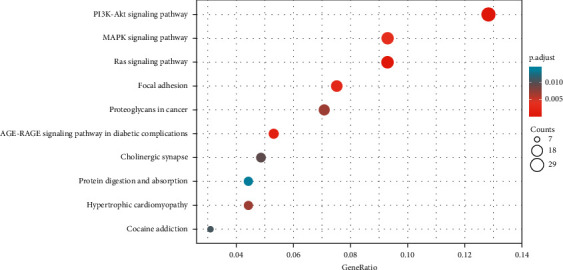
KEGG analysis of miR-149-3p target genes.

The shared genes are involved in the biological process, including regulation of cation transmembrane transport, regulation of metal ion transport, extracellular matrix organization, regulation of ion transmembrane transport, and morphogenesis of a branching structure; cellular components, including main axon, collagen-containing extracellular matrix, membrane raft, membrane microdomain, membrane region, and postsynaptic membrane; molecular functions, including growth factor binding, extracellular matrix structural constituent, platelet-derived growth factor binding, SMAD binding, extracellular matrix structural constituent conferring tensile strength (Figure 7). The miR-149-3p target genes are involved in PI3K-Akt signaling pathway, Ras signaling pathway, AGE-RAGE signaling pathway in diabetic complications, focal adhesion, MAPK signaling pathway, proteoglycans in cancer, hypertrophic cardiomyopathy, cholinergic synapse, cocaine addiction, and protein digestion and absorption (Figure 8).

### 3.6. The Correlation between miR-149-3p Expression and Immune Infiltration

**Figure 9:**
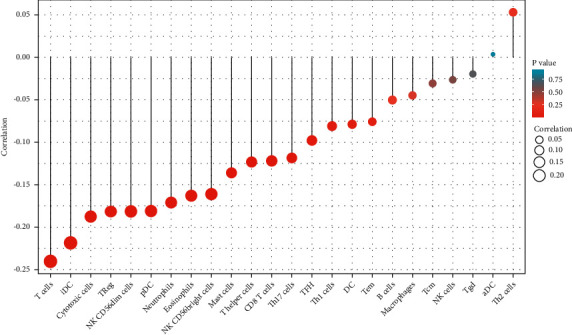
Correlation between miR-149-3p expression and 24 immune cells in UCEC (grouped comparison plots). Significance markers: ^*∗*^*P* < 0.05; ^*∗∗*^*P* < 0.01; ^*∗∗∗*^*P* < 0.001.

**Figure 10:**
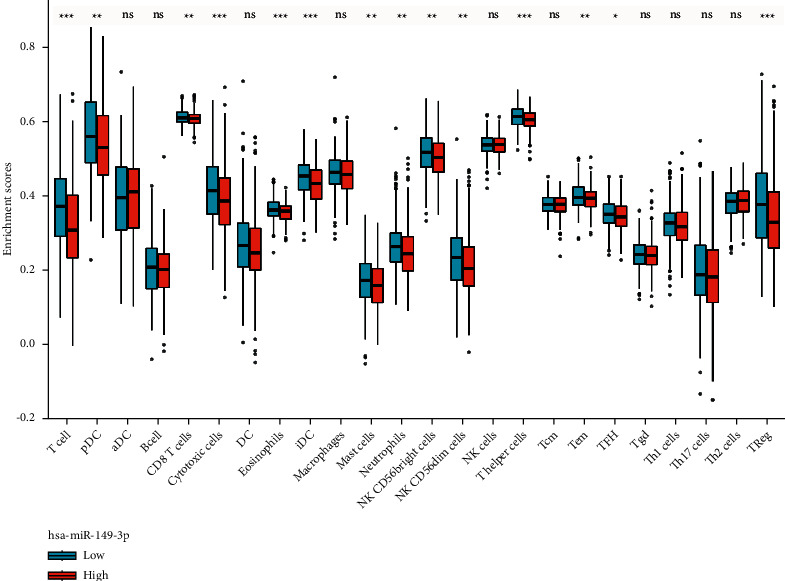
Correlation between miR-149-3p expression and 24 immune cells in UCEC (lollipop chart). The size of the dots indicates the absolute value of spearman *r*.

As shown in Figures 9 and 10 and Table 5, miR-149-3p expression was negatively correlated with that of CD8 T cells (*P*=0.005), cytotoxic cells (*P* < 0.001), eosinophils (*P* < 0.001), iDC (*P* < 0.001), mast cells (*P*=0.002), neutrophils (*P* < 0.001), NK CD56bright cells (*P* < 0.001), NK CD56dim cells (*P* < 0.001), pDC (*P* < 0.001), T cells (*P* < 0.001), T helper cells (*P*=0.004), TFH (*P*=0.024), Th17 cells (*P*=0.006), and Treg (*P* < 0.001).

### 3.7. Validation of miR-149-3p Expression in Cell Lines

**Figure 11:**
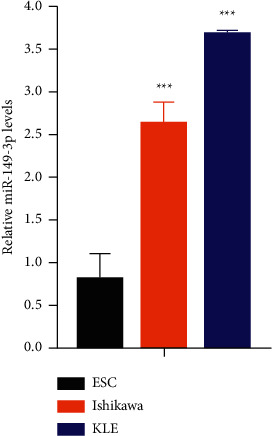
Expression of miR-149-3p in human endometriotic stromal cells (ESC), UCEC cell lines Ishikawa and KLE.

The expression of miR-149-3p in Ishikawa was significantly higher than that in ESC (2.608 ± 0.253 versus 0.874 ± 0.233, *P* < 0.001) (Figure 11). The expression of miR-149-3p in KLE was significantly higher than that in ESC (1.823 ± 0.055 versus 0.874 ± 0.233, *P* < 0.001) (Figure 11). These results suggested that miR-149-3p was significantly upregulated in UCEC cell lines compared with endometriotic stromal cells.

## 4. Discussion

The development of type I endometrial cancer is associated with continuous estrogen stimulation of the endometrium without progestin antagonism. The endometrium lacks progesterone antagonism, and continuous stimulation by estrogen will result in a prolonged state of hyperproliferation, which will further develop into endometrial cancer. MiRNAs regulate the cell cycle and cell differentiation and migration, which may also act as tumor suppressor genes or oncogenes during tumorigenesis and tumor development [32]. MiRNAs are also involved in the development of various cancers and are described in detail in UCEC. MiR-181c affects the growth of estrogen-dependent endometrial cancer cells by targeting PTEN, which may be an effective target for endometrial cancer therapy [33]. MiR-320a exerts antitumor effects on endometrial cancer by regulating IGF-1R, which can be used as a target for endometrial cancer gene therapy [34]. Downregulated miR-29b expression is associated with poor prognosis in endometrial cancer (EC) and contributes to the evaluation of EC prognosis [35]. miRNA-205 has potential clinical utility as a prognostic marker for endometrial cancer [36]. Therefore, detection of miR-149-3p is an ideal candidate for improving the early detection of UCEC.

In the present study, miR-149-3p was significantly correlated with age (*P* < 0.001), histological type (*P* < 0.001), histological grade (*P* < 0.001), tumor invasion (*P*=0.014), and radiation therapy (*P*=0.011). UCEC expressed more miR-149-3p than normal tissue, especially in patients with age (>60), histological type (mixed and serous), histologic grade (*G*2 and *G*3), tumor invasion (≥50%), or radiation therapy (yes). These phenomena suggested that miR-149-3p may be involved in tumor development and promote proliferation. And miR-149-3p was highly expressed in UCEC, and patients with high miR-149-3p expression had poorer OS (HR: 2.56; 95% CI: 1.64–4.00; *P* < 0.001), PFI (HR: 1.85; 95% CI: 1.29–2.65; *P*=0.001), and DSS (HR: 2.33; 95% CI: 1.37–3.99; *P*=0.002) of UCEC patients. Low expressions of miR-149-3p target genes, including ADCYAP1R1, CGNL1, CHST3, CYGB, DNAH9, ESR1, HHIP, HIC1, HOXD11, IGF1, INMT, LSP1, MTMR10, NFIC, PLCE1, RARA, SNTN, SPRYD3, and ZBTB7A, were associated with poor OS in UCEC. Lymphovascular space invasion (LVSI) has an independent influence on the poor prognosis of UCEC patients [37–39]. And this index can be used as an important reference indication for adjuvant therapy in endometrial cancer patients after surgery. The data of this study were not available for patients' LVSI data because they were obtained from TCGA UCEC. The correlation between miR-149-3p and LVSI in patients with endometrial cancer in the real world needs to be further investigated.

miR-149-3p inhibits bladder cancer proliferation, migration, and invasion targeting S100A4 [14]. Long noncoding RNA ARAP1-AS1 promotes cervical cancer progression through the regulation of miR-149-3p and POU2F2 [40]. miR-149-3p inhibits cell proliferation by targeting AKT2 in oral squamous cell carcinoma [41]. In this study, GO and KEGG analyses showed that the shared genes are involved in PI3K-Akt signaling pathway, Ras signaling pathway, AGE-RAGE signaling pathway in diabetic complications, focal adhesion, MAPK signaling pathway, proteoglycans in cancer, hypertrophic cardiomyopathy, cholinergic synapse, cocaine addiction, and protein digestion and absorption in UCEC. Phosphorylated proteome is involved in smooth muscle tumor growth, and biomimetic analysis revealed ten tumorigenic signaling pathways (FAS signaling pathway, p38 MAPK pathway, VEGF signaling pathway, Rho GTPase regulation of cytoskeleton, integrin signaling pathway, apoptosis signaling pathway, angiogenesis, gonadotropin-releasing hormone receptor pathway, ubiquitin-proteasome pathway, chemokines, and cytokine-mediated inflammatory signaling pathways) and four phosphoproteins (HSPA5, HSPB1, HSPD1, and PDRX2) involved in apoptosis and inhibition of cell survival [42]. The relationship between miR-149-3p and phosphoproteins needs further investigation.

Immune cell infiltration has emerged as a new indicator of prognosis for patients with different types of solid tumors [43]. There is heterogeneity in the immune response between tumors [44]. Immune infiltration in UCEC is currently a hot topic, and an understanding of immune infiltration will facilitate the development of immunotherapy for UCEC. The results of this study showed that miR-149-3p expression was correlated with infiltration of CD8 T cells, cytotoxic cells, eosinophils, iDC, mast cells, neutrophils, NK CD56bright cells, NK CD56dim cells, pDC, T cells, T helper cells, TFH, Th17 cells, and Treg in UCEC. It indicated that miR-149-3p may promote the function of CD8 T cells, cytotoxic cells, eosinophils, iDC, mast cells, neutrophils, NK CD56bright cells, NK CD56dim cells, pDC, T cells, T helper cells, TFH, Th17 cells, and Treg.

This study is limited to the analysis of UCEC tissues in the TCGA database, and further studies, including serological and clinical data and cellular experiments, are needed to confirm the results.

## 5. Conclusion

miR-149-3p was highly expressed in UCECs and correlated with poorer OS compared with normal tissues. The high miR-149-3p expression in UCEC was significantly associated with age (*P* < 0.001), histological type (*P* < 0.001), histological grade (*P* < 0.001), tumor invasion (*P*=0.014), and radiation therapy (*P*=0.011). miR-149-3p may be involved in the occurrence and development of UCEC via pathways, including PI3K-Akt signaling pathway, Ras signaling pathway, AGE-RAGE signaling pathway in diabetic complications, focal adhesion, and MAPK signaling pathway. Expression of miR-149-3p was correlated with immune infiltration in UCEC. This study partially elucidates the role of miR-149-3p in UCEC and provides a promising biomarker for prognosis and immunotherapy response in UCEC patients.

## Figures and Tables

**Table 1:** Correlation between miR-149-3p expression and clinical characteristics in UCEC.

Characteristic	Levels	Overall	Low expression of hsa-miR-149-3p	High expression of hsa-miR-149-3p	*P*
*n*		546	273	273	
Clinical stage, *n* (%)	Stage I	341 (62.5%)	182 (33.3%)	159 (29.1%)	0.208
	Stage II	49 (9%)	21 (3.8%)	28 (5.1%)	
	Stage III	128 (23.4%)	56 (10.3%)	72 (13.2%)	
	Stage IV	28 (5.1%)	14 (2.6%)	14 (2.6%)	
Primary therapy outcome, *n* (%)	PD	20 (4.2%)	7 (1.5%)	13 (2.7%)	0.080
	SD	6 (1.3%)	4 (0.8%)	2 (0.4%)	
	PR	12 (2.5%)	3 (0.6%)	9 (1.9%)	
	CR	437 (92%)	235 (49.5%)	202 (42.5%)	
Race, *n* (%)	Asian	20 (4%)	14 (2.8%)	6 (1.2%)	0.118
	Black or African American	109 (21.8%)	49 (9.8%)	60 (12%)	
	White	372 (74.3%)	185 (36.9%)	187 (37.3%)	
Age, *n* (%)	≤60	205 (37.8%)	125 (23%)	80 (14.7%)	<0.001
	>60	338 (62.2%)	148 (27.3%)	190 (35%)	
Weight, *n* (%)	≤80	240 (45.9%)	113 (21.6%)	127 (24.3%)	0.133
	>80	283 (54.1%)	153 (29.3%)	130 (24.9%)	
Height, *n* (%)	≤160	244 (47%)	127 (24.5%)	117 (22.5%)	0.616
	>160	275 (53%)	136 (26.2%)	139 (26.8%)	
BMI, *n* (%)	≤30	209 (40.6%)	104 (20.2%)	105 (20.4%)	0.743
	>30	306 (59.4%)	158 (30.7%)	148 (28.7%)	
Histological type, *n* (%)	Endometrioid	409 (74.9%)	233 (42.7%)	176 (32.2%)	<0.001
	Mixed	24 (4.4%)	9 (1.6%)	15 (2.7%)	
	Serous	113 (20.7%)	31 (5.7%)	82 (15%)	
Residual tumor, *n* (%)	*R*0	371 (90.9%)	192 (47.1%)	179 (43.9%)	0.432
	*R*1	22 (5.4%)	10 (2.5%)	12 (2.9%)	
	*R*2	15 (3.7%)	10 (2.5%)	5 (1.2%)	
Histologic grade, *n* (%)	*G*1	98 (18.3%)	72 (13.5%)	26 (4.9%)	<0.001
	*G*2	121 (22.6%)	78 (14.6%)	43 (8%)	
	*G*3	316 (59.1%)	121 (22.6%)	195 (36.4%)	
Tumor invasion (%), *n* (%)	<50	260 (55.3%)	152 (32.3%)	108 (23%)	0.014
	≥50	210 (44.7%)	98 (20.9%)	112 (23.8%)	
Menopause status, *n* (%)	Pre	34 (6.8%)	21 (4.2%)	13 (2.6%)	0.360
	Peri	17 (3.4%)	9 (1.8%)	8 (1.6%)	
	Post	449 (89.8%)	221 (44.2%)	228 (45.6%)	
Hormones therapy, *n* (%)	No	297 (86.6%)	158 (46.1%)	139 (40.5%)	0.806
	Yes	46 (13.4%)	23 (6.7%)	23 (6.7%)	
Diabetes, *n* (%)	No	323 (72.6%)	165 (37.1%)	158 (35.5%)	0.475
	Yes	122 (27.4%)	57 (12.8%)	65 (14.6%)	
Radiation therapy, *n* (%)	No	278 (53.4%)	157 (30.1%)	121 (23.2%)	0.011
	Yes	243 (46.6%)	109 (20.9%)	134 (25.7%)	
Surgical approach, *n* (%)	Minimally invasive	207 (39.6%)	113 (21.6%)	94 (18%)	0.197
	Open	316 (60.4%)	153 (29.3%)	163 (31.2%)	
Age, median (IQR)		64 (57, 71)	62 (55, 69)	66 (59, 72)	<0.001

**Table 2:** miR-149-3p expression correlated with clinical and pathological features (logistic regression analysis).

Characteristics	Total (*N*)	Odds ratio (OR)	*P* value
Clinical stage (stage III and stage IV versus stage I and stage II)	546	1.334 (0.919–1.940)	0.130
Primary therapy outcome (CR versus PD, SD, and PR)	475	0.501 (0.247–0.983)	0.048
Race (White versus Asian and Black or African American)	501	0.965 (0.646–1.441)	0.861
Age (>60 versus ≤60)	543	2.006 (1.411–2.863)	<0.001
Weight (>80 versus ≤80)	523	0.756 (0.535–1.067)	0.112
Height (>160 versus ≤160)	519	1.109 (0.786–1.567)	0.555
BMI (>30 versus ≤30)	515	0.928 (0.652–1.319)	0.676
Histological type (endometrioid versus mixed and serous)	546	0.311 (0.204–0.470)	<0.001
Residual tumor (*R*0 versus *R*1 and *R*2)	408	1.097 (0.557–2.182)	0.789
Histologic grade (*G*1 versus *G*2 and *G*3)	535	0.302 (0.183–0.485)	<0.001
Tumor invasion (%) (≥50 versus <50)	470	1.608 (1.116–2.324)	0.011
Menopause status (pre versus peri and post)	500	0.603 (0.288–1.219)	0.166
Hormones therapy (yes versus no)	343	1.137 (0.609–2.123)	0.686
Diabetes (yes versus no)	445	1.191 (0.785–1.810)	0.412
Radiation therapy (yes versus no)	521	1.595 (1.129–2.259)	0.008
Surgical approach (open versus minimally invasive)	523	1.281 (0.902–1.822)	0.168

**Table 3:** Univariate and multivariate Cox regression analysis of OS and clinical features in UCEC patients.

Characteristics	Total (*N*)	Univariate analysis		Multivariate analysis	
		HR (95% CI)	*P* value	HR (95% CI)	*P* value
Clinical stage (stage I and stage II versus stage III and stage IV)	545	3.774 (2.484–5.732)	<0.001	3.912 (1.711–8.946)	0.001
Primary therapy outcome (PD, SD, and PR versus CR)	475	0.125 (0.075–0.208)	<0.001	0.299 (0.130–0.689)	0.005
Age (≤60 versus >60)	543	2.002 (1.236–3.242)	0.005	1.944 (0.868–4.355)	0.106
Weight (≤80 versus >80)	522	1.032 (0.676–1.576)	0.885		
Height (≤160 versus >160)	518	1.115 (0.728–1.708)	0.616		
BMI (≤30 versus >30)	514	0.942 (0.616–1.442)	0.784		
Histological type (mixed and serous versus endometrioid)	545	0.366 (0.241–0.557)	<0.001	1.285 (0.615–2.685)	0.505
Residual tumor (*R*0 versus *R*1 and *R*2)	407	3.081 (1.725–5.503)	<0.001	1.951 (0.811–4.694)	0.136
Histologic grade (*G*1 versus *G*2 and *G*3)	534	22.795 (3.172–163.794)	0.002	156521500.831 (0.000-Inf)	0.997
Tumor invasion (%) (<50 versus ≥50)	469	3.124 (1.909–5.112)	<0.001	1.111 (0.532–2.320)	0.779
Menopause status (peri and post versus pre)	499	1.070 (0.466–2.457)	0.874		
Hormones therapy (no versus yes)	343	0.872 (0.412–1.845)	0.72		
Diabetes (no versus yes)	445	1.188 (0.733–1.925)	0.485		
Radiation therapy (no versus yes)	521	0.586 (0.375–0.914)	0.018	0.320 (0.165–0.622)	<0.001
Surgical approach (minimally invasive versus open)	522	0.731 (0.474–1.127)	0.156		
hsa-miR-149-3p (low versus high)	545	2.559 (1.637–4.002)	<0.001	2.393 (1.161–4.935)	0.018

**Table 4:** The relationship between miR-149-3p target gene expression level and prognosis.

Gene name	HR	CI	*P* value
PLCE1	0.49	0.318–0.754	0.001
DNAH9	0.66	0.437–0.997	0.048
ADCYAP1R1	0.655	0.433–0.991	0.045
RARA	0.622	0.408–0.949	0.027
IGF1	0.62	0.409–0.939	0.024
CYGB	0.597	0.394–0.905	0.015
CHST3	0.638	0.421–0.967	0.034
HIC1	0.623	0.412–0.943	0.025
INMT	0.603	0.397–0.916	0.018
CGNL1	0.616	0.406–0.934	0.022
NFIC	0.499	0.326–0.765	0.001
ESR1	0.519	0.339–0.795	0.003
HHIP	0.659	0.437–0.993	0.046
HOXD11	0.65	0.430–0.984	0.042
ZBTB7A	0.613	0.404–0.932	0.022
SPRYD3	0.641	0.423–0.972	0.036
MTMR10	0.617	0.406–0.936	0.023
LSP1	0.503	0.328–0.771	0.002
SNTN	0.613	0.406–0.925	0.020

**Table 5:** Expression of miR-149-3p in relation to immune cells (spearman method).

Gene name	Cell type	Correlation coefficient (spearman)	*P* value (spearman)
hsa-miR-149-3p	aDC	0.004	0.933
hsa-miR-149-3p	B cells	−0.05	0.244
hsa-miR-149-3p	CD8 T cells	−0.122	0.005
hsa-miR-149-3p	Cytotoxic cells	−0.187	<0.001
hsa-miR-149-3p	DC	−0.079	0.068
hsa-miR-149-3p	Eosinophils	−0.163	<0.001
hsa-miR-149-3p	iDC	−0.218	<0.001
hsa-miR-149-3p	Macrophages	−0.045	0.298
hsa-miR-149-3p	Mast cells	−0.136	0.002
hsa-miR-149-3p	Neutrophils	−0.171	<0.001
hsa-miR-149-3p	NK CD56bright cells	−0.161	<0.001
hsa-miR-149-3p	NK CD56dim cells	−0.181	<0.001
hsa-miR-149-3p	NK cells	−0.027	0.539
hsa-miR-149-3p	pDC	−0.181	<0.001
hsa-miR-149-3p	T cells	−0.24	<0.001
hsa-miR-149-3p	T helper cells	−0.123	0.004
hsa-miR-149-3p	Tcm	−0.031	0.476
hsa-miR-149-3p	Tem	−0.076	0.08
hsa-miR-149-3p	TFH	−0.098	0.024
hsa-miR-149-3p	Tgd	−0.019	0.653
hsa-miR-149-3p	Th1 cells	−0.081	0.062
hsa-miR-149-3p	Th17 cells	−0.118	0.006
hsa-miR-149-3p	Th2 cells	0.053	0.221
hsa-miR-149-3p	Treg	−0.182	<0.001

## Data Availability

All data generated or analyzed during this study are included in this article. The datasets generated in this study are available from TCGA that provides free resources.
